# Technical Note – Lateral Approach to the Lumbar Spine for the Removal of Interbody Cages

**DOI:** 10.7759/cureus.268

**Published:** 2015-05-11

**Authors:** Marc Moisi, Jeni Page, David Paulson, Rod J Oskouian

**Affiliations:** 1 Neurosurgery, Swedish Neuroscience Institute; 2 Department of Neurosurgery, Swedish Neuroscience Institute; 3 Neurosurgey, Swedish Neuroscience Institute

**Keywords:** lateral interbody fusion, pseudoarthrosis, cage removal, interbody cage migration, revision surgery

## Abstract

Revision surgery to address the migration or fracture of a lumbar interbody cage can be technically challenging. Scar tissue and fibrosis, among other anatomic barriers, can make removal of the cage a complicated procedure, potentially increasing postoperative pain as well as the probability of neurologic deficits. Use of the lateral surgical technique for removal of the cage can avoid these potential complications. In this case report, we describe the removal of interbody cages through a lateral approach in three patients without the necessity of additional posterior hardware revision.

## Introduction

A lateral interbody fusion is a minimally invasive, transpsoas, retroperitoneal surgical approach to the thoracic and lumbar intervertebral disc space [1]. Use of this technique in revision surgery can reduce the risk of complications more common with traditional approaches, both anterior and posterior [2-3]. Particularly in cases of interbody cage migration, cage fracture, or those with failure to fuse, this technique allows direct access to the cage while avoiding scar tissue and epidural fibrosis. However, few studies have been published discussing this approach for cage removal not requiring posterior hardware revision [3-4]. In this case report, we discuss our experience with the lateral retrieval of interbody cages that have migrated, fractured, or not fused, and subsequently required surgical intervention but did not require posterior hardware revision.

## Technical report

Approval for this study was obtained from the Swedish Institutional Review Board (IRB #FWA00000544). Informed patient consent was obtained from all subjects.

### Representative cases

Presentation

**Figure 1:**
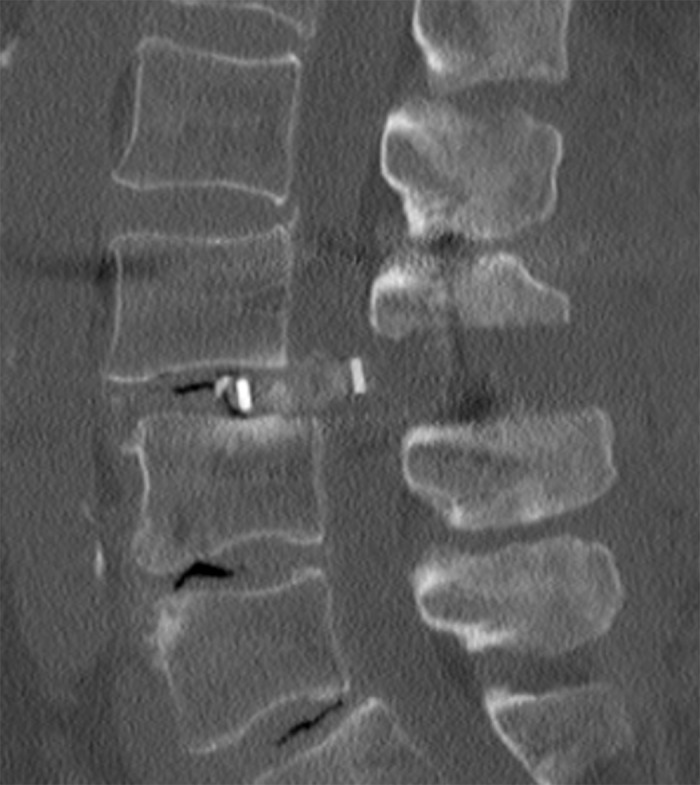
Cage Migration Computed tomography (CT) demonstrating migration and displacement of the interbody cage into the spinal canal causing severe central canal stenosis.

The first patient reviewed was a 71-year-old female who fell six weeks postoperatively following a transforaminal lumbar interbody fusion (TLIF) at L3-4. Subsequently, she developed lumbar and radicular pain. Computed tomography (CT) demonstrated displacement of the interbody cage into the spinal canal causing severe central canal stenosis (Figure 1).

**Figure 2:**
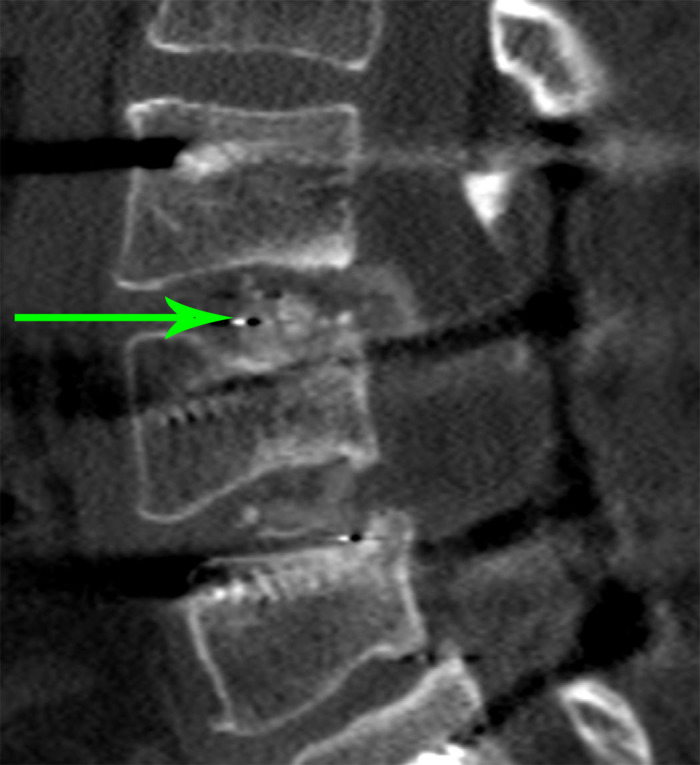
Cage Fracture and Migration CT imaging reveals a fracture of the interbody cage and its subsequent migration into the spinal cord.

In the second patient case, a 52-year-old female was evaluated in clinic with symptoms of significant neurogenic claudication, radicular pain, and lower extremity weakness two years status post posterior lumbar interbody fusion at L3-4. CT imaging revealed a fracture of the interbody cage and its subsequent migration into the spinal cord (Figure 2).

The third patient reviewed was a 50-year-old female who presented for evaluation of increasing back pain one-year status-post L2 to L5 fusion. CT imaging demonstrated with pseudoarthrosis of the (TLIF) interbody cage at the L3-4 level with subsidence causing significant lumbar stenosis.

Operative Technique

**Figure 3:**
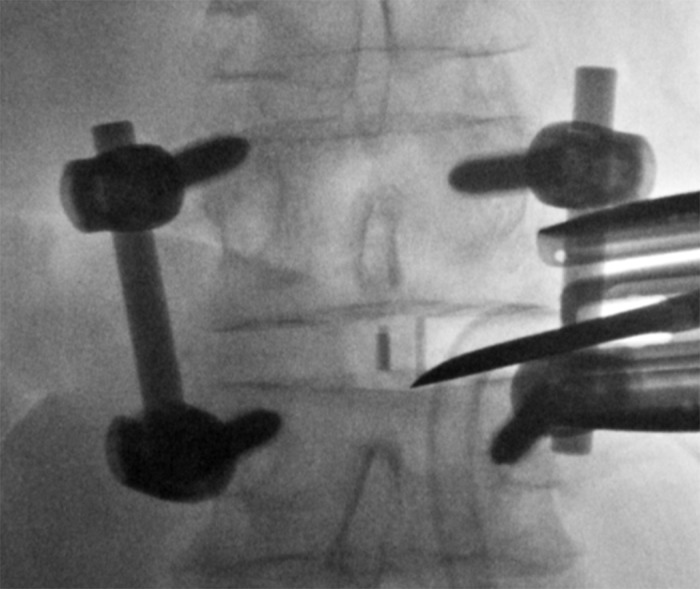
Loosening of the Cage An osteotome can be seen approaching the disc space laterally along the inferior end plate. This is done in order to loosen the cage.

Once docked on the lateral disc space, we used an osteotome to go above and below the cage as well as along the end plate (Figure 3).

**Figure 4:**
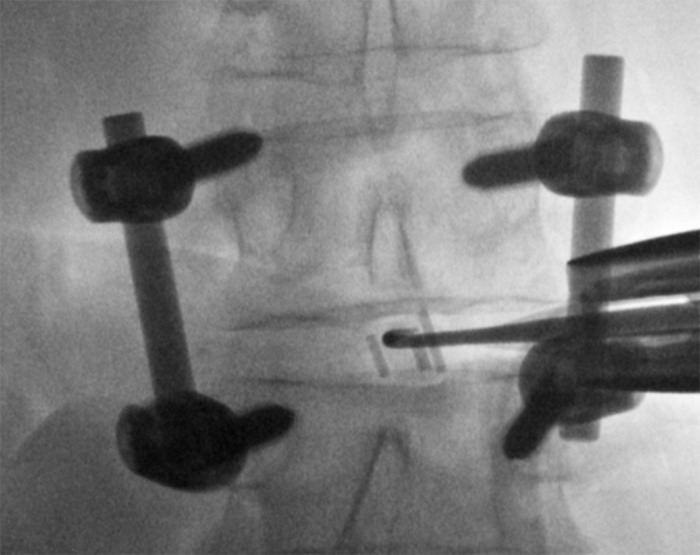
Cage Retrieval After the cage is loosened, a hook or pituitary with teeth is used to retrieve the material.

This was completed in order to loosen and remove the interbody cage. In some instances, a straight and a curved curette were used to loosen the cage from scar tissue and the end plates. Once the cage was properly loosened, a hook or pituitary with teeth was inserted to grab the cage allowing it to be withdrawn from the interspace (Figure 4).

In all three cases, once the cages were confirmed by fluoroscopy to have been removed, the disc space was then prepared in the usual fashion, a new cage was inserted, and a lateral interbody fusion was performed. Although there are some constraints placed on the size of the new cage since there is already posterior fixation, the goal of this revision surgery is not to maintain a significant amount of added indirect decompression, but to provide stabilization to the anterior column.

## Discussion

Revision surgery can be technically challenging with epidural fibrosis and scarring from prior surgical interventions making exposures complex and leading to increased risk of an incidental durotomy and nerve root injury [5-6]. However, removal of failed hardware is frequently necessary when there is subsequent compression of neural elements and spinal cord resulting in lower extremity pain and weakness [7]. Interbody cages can migrate or a fractured piece can displace in the anterior, posterior, or lateral directions. Historically, removal of these cages has been completed in the traditional approaches through an anterior or posterior incision.

The anterior approach for removal of an interbody cage poses similar risks to those of the initial surgery, as well as possible complications related to fibrosis. It can be an appealing technique as it preserves the posterior paravertebral muscles and ligaments, but carries multiple risks, including retrograde ejaculation, ileus, abdominal muscle weakness, and lymphocele [8-11]. Furthermore, the anterior approach, due to its close proximity to the great vessels, can lead to significant vascular injury [11-12]. Vascular injury poses particularly high risks with revision surgery due to the presence of retroperitoneal fibrosis [12-14]. Even with the assistance of access surgeons, one study found vascular injury occurred in 57% of their cases [15]. This same study concluded both blood loss and length of stay were higher with anterior revision surgery. Despite these risks, cage displacement location or patient anatomy may warrant the anterior approach, and while the cage can be successfully revised with this technique, there is frequently a need for more extensive surgery. Glassman, et al. described an anterior technique for removal of the cage, which was successful [16]. However, this procedure ultimately required a partial vertebral body resection. Oh, et al. similarly used the anterior approach for removal and replacement of the interbody cage, but in this particular study, the patient also required a posterior approach for revision of the segmental instrumentation [17].

Removal of the interbody cage from a posterior technique can be difficult for multiple reasons. Epidural fibrosis from prior surgery can alter the natural planes and anatomic landmarks making location identification challenging [6]. This in turn can lead to increased dural retraction and nerve root mobilization with the exposure [18-19]. Additionally, in cases where stripping of the paravertebral musculature is necessary, the retraumatization to the posterior structures can increase risk of significant postoperative myofascial pain, bleeding, infection, and nerve injury up to 15-30% [5, 19]. To avoid these complications, some studies have evaluated implementing the posterior minimally invasive technique for revision [6]. However, Selznick, et al. suggests this approach, particularly for revision of a lumbar interbody fusion, harbors a significantly higher risk of a cerebrospinal fluid (CSF) leak [6]. Furthermore, these studies did not examine revision of the interbody cage alone.

The introduction of the lateral approach provided an access to the anterior lumbar spine without the need of an access surgeon [11]. While the technique has been successfully utilized for multiple spinal conditions, the approach has gained popularity in revision surgery because it involves navigating virgin tissue and thus avoiding the complications associated with traditional exposures [11]. Besides the ease and direct minimally invasive access of the approach, the technique increases safety to an area that has been previously disturbed with past surgical attempts [11].

The lateral approach is not without complications. Due to the nature of the muscle splitting approach through the psoas, as well as the close proximity to the lumbar plexus, transient nerve injury can occur causing temporary pain, weakness, or numbness [11, 20-23]. If injured, these nerves can cause detrimental consequences, making the use of neurophysiologic monitoring a necessity [24-25]. Hip flexor weakness and transient anterior thigh sensory changes have been reported in 28% and 18% of the patients, respectively, generally resolving in the early postoperative period [26]. For this reason, a thorough understanding of the lateral anatomy, as well as a good understanding of the technique, will help to avoid these types of complications [27-28].

Also of consideration is when the technique can be effectively used. Interbody cage position and displacement can play a crucial role in this process. This technique can be used to retrieve cages previously placed through posterior, anterior, and lateral approaches, but a careful understanding of the anatomy and pitfalls is necessary. When the cage is too posterior with significant migration into the canal, there is an increased risk of pushing it further posterior and causing a cerebrospinal fluid (CSF) leak. Similarly, if the cage is too anterior, the great vessels are at risk to be injured. Close proximity to the great vessels does not exclude the lateral approach but does warrant close evaluation. CT imaging as well as lateral/anteroposterior (AP) radiographs completed prior to proceeding with surgery can help to assure the cage is accessible [11]. Furthermore, the lateral approach may not be feasible depending on the anatomy of the patient. In patients with a high iliac crest, the crest impedes access to the disc space. Thus, the L5-S1 level is not accessible, and on occasion, the L4-5 level cannot be reached [26, 28].

## Conclusions

When the decision to remove an interbody cage is made in which a posterior hardware revision is not indicated, use of the lateral technique is an option that provides great results with all the cases discussed leading to fusion and resolution of preoperative symptoms. Surgeons considering this approach should have a good understanding of the lateral anatomy as well as recognize indications for this procedure with revision surgery. Patient cases should be examined on an individualized basis with special consideration given to cage location and patient anatomy.
